# Editorial Note: Regulation of Vapor Pressure Deficit by Greenhouse Micro-Fog Systems Improved Growth and Productivity of Tomato via Enhancing Photosynthesis during Summer Season

**DOI:** 10.1371/journal.pone.0351610

**Published:** 2026-06-15

**Authors:** The PLOS One Editors

Following publication of this article [1], concerns were raised regarding the origins of the data in [1].

The first author stated that the environmental data presented in Fig 2 originates from a different institution and was inadvertently included in [1]. Please view the correct Fig 2 below.

In the Effect of the micro-fog system on greenhouse environment subsection of the Results, there is an error in the third and fourth sentences of the second paragraph. The correct sentences are: The mean values of temperature and VPD during midday period was 34.4°C and 2.8 KPa in the control site, whereas it was reduced to 28.6°C and 0.8KPa in the micro-fog applied site, respectively. Air relative humidity was 49.4% in the control site and increased to 80.6% in the compartment of micro-fog treatment.

In the Effect of the micro-fog system on greenhouse environment subsection of the Results, there is an error in the first and second sentences of the third paragraph. The correct sentences are: In addition, micro-fog also reduced the fluctuation in meteorological variables, especially the relative humidity and VPD. The stand deviation of the relative humidity and VPD was 5.16 and 0.36 in the control compartment, whereas it was reduced to 4.66 and 0.19 in the micro-fog applied site, respectively.

PLOS is unable to verify the provenance of the data in [1] due to conflicting recommendations from the relevant institutions.

Additionally, author DZ stated that the dimensions of the greenhouse used in this study were incorrectly reported in both Fig 1B and in the second sentence of the second paragraph of the Experimental site and greenhouse specification subsection of the Materials and Methods section. The correct sentence is: The greenhouse-dimensions were 24m in length and 19.2m in width, the height was 5.3m from ground to the gutter. Please view the correct Fig 1 with the corrected measurements below.

The raw data underlying Figs 2-5 and Tables 1-3 are not included in the original submission. The authors have provided the data as S1 File. With this notice, all relevant data are now provided.

The *PLOS One* Editors issue this Editorial Note to make readers aware of the above information and concerns.

**Fig 1:**
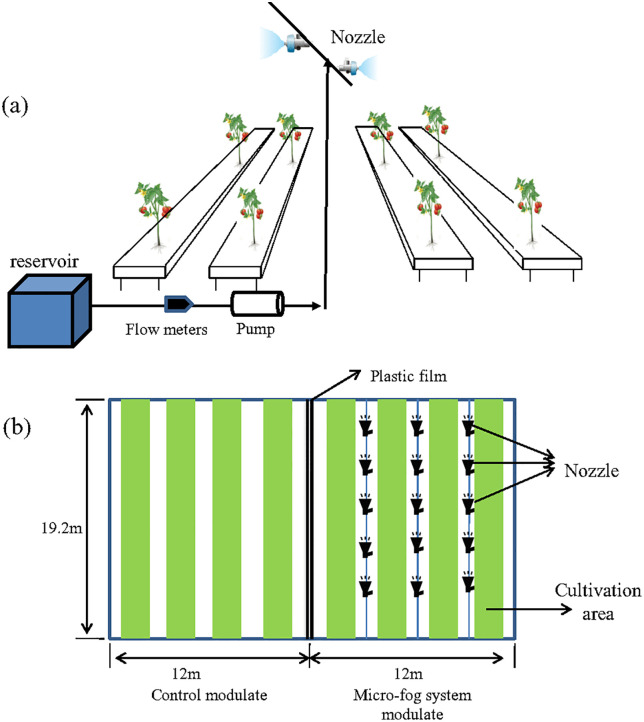
Scheme of the greenhouse and micro-fog system installation. **(a)** Schematic description of the micro-fog system, (b) top view of the greenhouse compartments.

**Fig 2:**
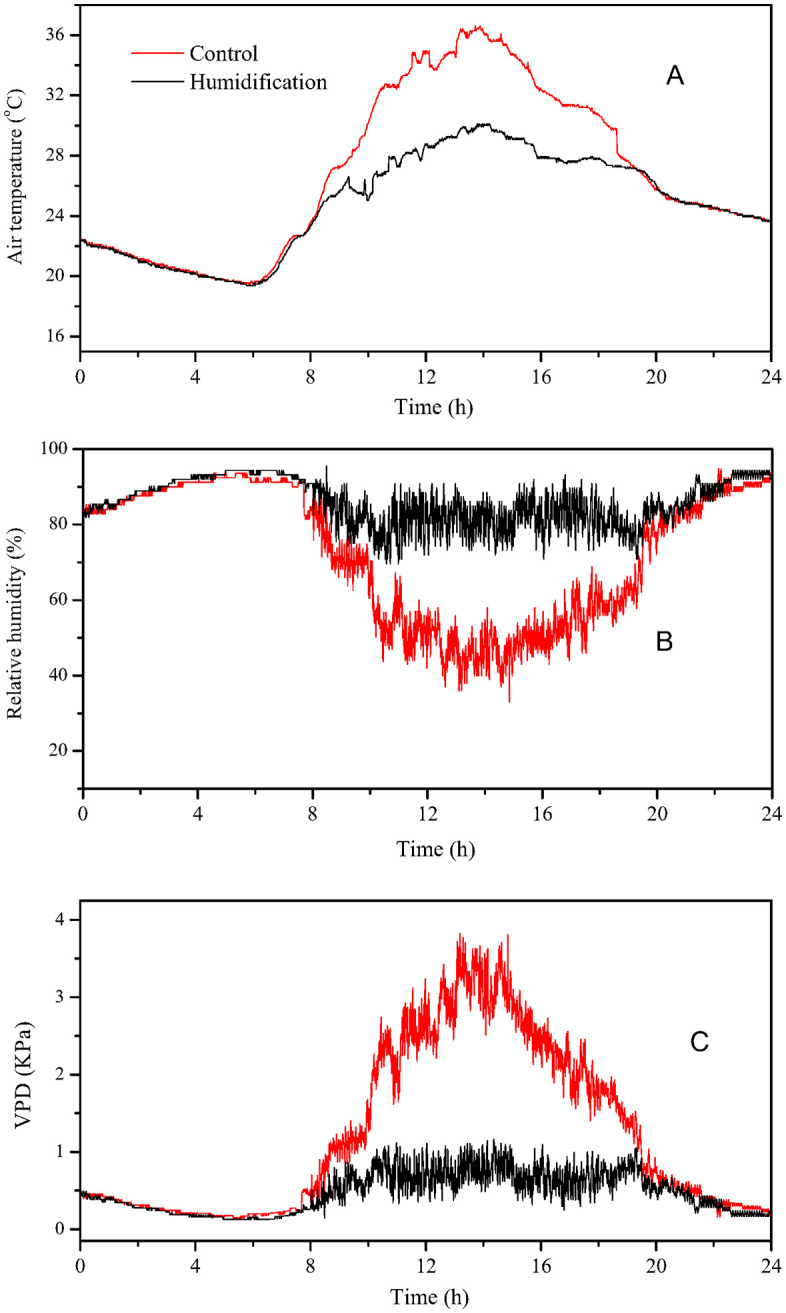
Comparison of typical diurnal variation of greenhouse environment. Air temperature, relatively humidity, VPD between control and humidification treatment were measured at DAT 34 (19 Aug).

## Supporting information

S1 File Quantitative data underlying Figs 2–5 & Tables 1–3. (XLSX)
